# Epidemiological characteristics and spatiotemporal patterns of scrub typhus in Fujian province during 2012–2020

**DOI:** 10.1371/journal.pntd.0010278

**Published:** 2022-09-29

**Authors:** Li Qian, Yong Wang, Xianyu Wei, Ping Liu, Ricardo J. Soares Magalhaes, Quan Qian, Hong Peng, Liang Wen, Yuanyong Xu, Hailong Sun, Wenwu Yin, Wenyi Zhang

**Affiliations:** 1 Department of Health Statistics, College of Preventive Medicine, Army Medical University, Chongqing, China; 2 Chinese PLA Center for Disease Control and Prevention, Beijing, China; 3 Department of Epidemiology and Biostatistics, School of Public Health, Anhui Medical University, Hefei, China; 4 Department of General Practice, Chinese PLA General Hospital-Sixth Medical Center, Beijing, China; 5 Spatial Epidemiology Laboratory, School of Veterinary Science, The University of Queensland, Brisbane, Australia; 6 Child Health Research Center, The University of Queensland, Brisbane, Australia; 7 Chinese Center for Disease Control and Prevention, Beijing, China; University of Oxford, UNITED KINGDOM

## Abstract

**Background:**

Scrub typhus has become a serious public health concern in the Asia-Pacific region including China. There were new natural foci continuously recognized and dramatically increased reported cases in mainland China. However, the epidemiological characteristics and spatiotemporal patterns of scrub typhus in Fujian province have yet to be investigated.

**Objective:**

This study proposes to explore demographic characteristics and spatiotemporal dynamics of scrub typhus cases in Fujian province, and to detect high-risk regions between January 2012 and December 2020 at county/district scale and thereby help in devising public health strategies to improve scrub typhus prevention and control measures.

**Method:**

Monthly cases of scrub typhus reported at the county level in Fujian province during 2012–2020 were collected from the National Notifiable Disease Surveillance System. Time-series analyses, spatial autocorrelation analyses and space-time scan statistics were applied to identify and visualize the spatiotemporal patterns of scrub typhus cases in Fujian province. The demographic differences of scrub typhus cases from high-risk and low-risk counties in Fujian province were also compared.

**Results:**

A total of 11,859 scrub typhus cases reported in 87 counties from Fujian province were analyzed and the incidence showed an increasing trend from 2012 (2.31 per 100,000) to 2020 (3.20 per 100,000) with a peak in 2018 (4.59 per 100,000). There existed two seasonal peaks in June-July and September-October every year in Fujian province. A significant positive spatial autocorrelation of scrub typhus incidence in Fujian province was observed with Moran’s I values ranging from 0.258 to 0.471 (P<0.001). Several distinct spatiotemporal clusters mainly concentrated in north and southern parts of Fujian province. Compared to low-risk regions, a greater proportion of cases were female, farmer, and older residents in high-risk counties.

**Conclusions:**

These results demonstrate a clear spatiotemporal heterogeneity of scrub typhus cases in Fujian province, and provide the evidence in directing future researches on risk factors and effectively assist local health authorities in the refinement of public health interventions against scrub typhus transmission in the high risk regions.

## Author summary

Scrub typhus is a vector-borne zoonotic disease caused by *Orientia tsutsugamushi* and is popular in the Asia-Pacific area. Nowadays scrub typhus has been recognized as a considerable burden on public health in Fujian province. We explored the epidemiological characteristics, spatiotemporal patterns and diffusion characteristics of scrub typhus, and detected high-risk regions at the county level in Fujian province between January 2012 and December 2020. Our results indicated that the majority of cases were reported in June-July and September-October and that that middle aged and elderly people were more prone to infection every year in Fujian province. The spatial autocorrelation analysis revealed clustering in geographic distribution of cases and several distinct spatiotemporal clusters were identified in north and southern parts of Fujian province. Compared with cases from low-risk areas, a higher proportion of cases were female, farmer, and older residents in high-risk counties.

## Introduction

Scrub typhus is a life-threatening infectious disease caused by the bites of chigger mites that carry gram-negative obligately intracellular bacillus *Orientia tsutsugamushi*. It is estimated that scrub typhus results in one million cases annually and more than one billion people are exposed to the risk globally [1–3]. The disease is so fatal that patients without medical treatment has a median mortality of 6.0% [1]. The epidemic area of scrub typhus is mainly distributed from the Russian Far East in the north to Australia in the south, Japan in the east and Pakistan in the west, which is called the “tsutsugamushi triangle” in the traditional sense. While occasional cases were reported from United Arab Emirates (UAE), Chile and Africa neighboring the Asia Pacific region, and this suggested gradually expanding influence of scrub typhus [3–6].

Scrub typhus has become a more serious threat than previously known in China. In particularly, there was an alarming rise of annual incidence rate increasing from 0.09/100,000 in 2006 to 1.6/100,000 population in 2016 (>16-fold) and significant expansion of geographical distribution throughout mainland China during past two decades. Almost all the cases (99.7%) from 2006 to 2016 are concentrated in 15 provinces that are categorized into three epidemiologic regions including southeast China (21°–31°N, 105°–125°E), southwest parts (21°–31°N, 95°–105°E) and middle-east areas (31°–41°N, 105°–125°E) [7]. To be more specific, scrub typhus cases can be mainly grouped into 6 clusters on a national scale except occasional cases reported from Hunan Province and Tibet Autonomous Region. The important primary cluster, containing provinces of Guangdong, southern Fujian, Jiangxi, and Guangxi, is dispersed throughout the south and southeast of China [8]. The prevalence season of scrub typhus varies significantly from the north to the south and southwest districts. The seasonal onset of the disease occurs between May and July in both the south and southwest and the number of cases shows a sustained peak from June to September for the south and a single large peak in July or August for the southwest. By contrast, scrub typhus season in the north extends from September to December [9].

The provinces of Yunnan and Guangdong had the most annual average incidence of 6.18/100,000 and 3.05/100,000 people respectively based on recorded scrub typhus cases from 2006 to 2018[10]. A total of 57 death cases of scrub typhus reported from 2006 to 2016 demonstrated that the first and second highest number of deaths are still these two provinces with 28 cases in Guangdong and 11 cases in Yunnan [11]. In addition to the southeast coastal area of Guangdong province and the southwest border region of Yunnan province, both of which are traditionally considered as the main epidemic area for tsutsugamushi disease in China, the most severely affected place is Fujian province. Its annual average incidence rate and number of deaths from 2006 to 2016 are all highest compared to the other districts. However, there are still lack in more in-depth systematic research and plenty of attention paid to the epidemic status of tsutsugamushi disease in Fujian. High heterogeneity of incidence in demography, space and time can be preliminarily observed from the morbidity data of scrub typhus in this province. This has ascertained the necessity to thoroughly understand the demographic and spatiotemporal distribution patterns of scrub typhus, recognizing the potential infection risk zones and higher-risk individuals in order for better disease prevention and control in Fujian.

Spatiotemporal analysis techniques have been widely employed to investigate infectious diseases [12–15]. On the other hand, knowledge of the spatial-temporal patterns of scrub typhus in Fujian province is essential to minimize the public health burden imposed by the severe infection because the potential disease risks in the area is currently underestimated and studies remain poorly conducted. To address these gaps in knowledge and depict the profile of scrub typhus comprehensively and meticulously, our study was designed to describe and quantify the scrub typhus epidemic status throughout Fujian province, explore the presence of spatial and temporal trends in disease incidence, identify and evaluate the spatiotemporal clusters of scrub typhus cases at the county level. Further, this work will provide evidence-based suggestions for local health departments to determine the optimal allocation of limited resources and implement preemptive public health interventions such as enhanced targeted surveillance.

## Materials and methods

### Ethics statement

This study was approved by the Ethics Committee of Chinese PLA Center for Disease Control and Prevention and Chinese Center for Disease Control and Prevention. All the data analyzed in this study were de-identified to protect patient confidentiality. And the aggregate data were used to analyze in our study.

### Data collection and management

In China, scrub typhus cases are required by law to report to the Chinese Center for Disease Control and Prevention through Chinese National Notifiable Infectious Disease Reporting Information System (CNNDS). We collected the data on scrub typhus cases from January 2012 through December 2020 from CNNDS including information on gender, age, occupation, date of onset and diagnosis, case category, and residential address, etc.

All scrub typhus cases were classified as suspected, probable (clinically diagnosed) or confirmed (laboratory confirmed) in accordance with the unified criteria issued by the China CDC. Suspected cases are defined as an individual with: a) Epidemiological exposure history within 3 weeks prior to illness onset and; b) Fever with skin rash or lymphadenopathy. Patients who were presented fever, skin rash and lymphadenopathy without clear field exposure history should also be considered as suspected cases. Probable cases are suspected cases with specific eschar or ulcer manifested. Confirmed cases are clinically diagnosed patients meeting at least one positive result among the following laboratory tests: a) agglutination titer ≥ 1:160 in the Weil–Felix test using the OX-K strain of Proteus mirabilis, b) a 4-fold or more rise of serum IgG antibody titers between acute and convalescent sera using the indirect immunofluorescence antibody assay, c) detection of *O*. *tsutsugamushi* by polymerase chain reaction in clinical specimens, or d) isolation of *O*. *tsutsugamushi* from clinical specimens[7,16]. The annual demographic data at the county level in Fujian province during 2012–2020 were collected from Fujian provincial Bureau of Statistics. For the purpose of conducting a GIS-based analysis on the spatiotemporal distribution of scrub typhus, the 9-year data set of cases was aggregated at the county level. The county-level vector map at 1:1,000,000 scale was obtained from the National Catalogue Service For Geographic Information. All scrub typhus cases were geocoded and matched to the county-level layers of the polygon which are integrated with demographic information according to administrative codes using the ArcGIS software (version 10.5, ESRI, Redlands, CA) [17].

### Demographic distribution analysis

For the purpose of determining whether the incidence and distribution of cases grouped by the epidemiologic characteristics were different, demographic data were gathered for each scrub typhus case and a chi-square (χ^2^) test was performed by using SPSS software (version 25, IBM Corp., Armonk, NY, USA). P-value < 0.05 were considered statistically significant [18].

### Temporal analysis of scrub typhus incidence

Monthly scrub typhus cases at the county-level in Fujian provide were aggregated for time series analysis. A seasonal-trend decomposition of time series was applied to explore the various characteristics of periodicity and seasonality in incidence over a 9-year period by R software (Version 4.1, Lucent Technologies Bell Laboratories, Auckland, New Zealand) [19,20]. Seasonal and Trend decomposition using Loess (STL, Loess stands for locally estimated scatterplot smoothing) is a versatile and robust filtering method to decompose time series into trend component, seasonal component and random component for the reason that occasional anomalous observations do not affect the estimation of trend and seasonal components. In the present study, trend component that is low-frequency, long-term and relatively stable variable in data implies overall changes of 9 years in scrub typhus morbidity rate and seasonal component which is fixed time variation close to the season means periodic fluctuations of incidence on a yearly cycle. The residual term, namely random part which is an only tiny irregular fraction of the time series data theoretically, belongs to noises, outliers or something unexplainable in the incidence data.

The STL was implemented through a two-layer loop: (1) The inner loop will alternately update trends and seasonal components which can be done by subtracting the trend estimate of the previous round from the original time sequence. The time series is then divided into periodic subseries (for example, if annual data is sorted by month, there will be 12 periodic subseries: all January as one sequence, all February as one sequence, and so on). The cyclic sub-sequence is first smoothed by loess and then processed by a low-pass filter so the seasonal component is the smooth periodic subsequence minus the result of low pass filter. The trend can subsequently be obtained by smoothing the result, the raw data subtracting the seasonal component, by loess and what is left over is the residual. (2) Each iteration of the outer loop includes a nested inner loop and the calculation of robustness weights that are assigned to each data point based on the size of the residual portion obtained in the previous inner loop. Data points with very large residuals will have a very small or zero weight and these weights will be employed in the next inner cycle to reduce the effect of temporary or quirky points on trend and season terms. The two loops above were realized using the STL function of the feasts package in R version 4.1.0. Seasonal and trend component in time series of scrub typhus incidence were showed in order to better understand occurrence regularity of scrub typhus on different time scales.

### Spatial and spatiotemporal cluster analysis

Changes in the spatial distribution of incidence between 2012 and 2020were mapped using ArcGIS software. To reveal the difference, annualized incidences of scrub typhus per 100,000 at each administrative region over the nine-year period were calculated from the total number of cases in a county divided by resident population within the county. All counties were grouped into four categories: non-endemic area, low endemic area with annual incidence between 0/100,000 and 5/100,000, medium endemic area with the incidence between 5/100,000 and 10/100,000, high endemic area with the incidence between 10/100,000 and 20/100,000 and extra high endemic area with the incidence over 20/100,000[21].

There are three phases to conduct space-time cluster analysis. Firstly, Global Moran’s I for global indication of spatial autocorrelation assesses whether there was the similarity of annual incidence rate in spatial adjacent regions. The value ranging from -1 to 1 was calculated based on Z-test (P≤0.05). The closer it approaches 1, the more aggregated the whole incidence is while the more it close to -1, the more dispersed the distribution is. Moran’s I around 0 implies global random distribution. Secondly, Local Moran’s I for local indication of spatial autocorrelation (LISA) measures the similarity or difference between the incidence of the given county unit and those of surrounding counties. LISA was adopted to identify significant hotspots (High-High), cold spots (Low-Low), and outliers (High-Low and Low-High) of scrub typhus. The significance level of clusters was determined by a Z score generated by comparison of the Local Moran’s I statistic for the average incidence in each county. A high positive Z score represented that the surroundings had spatial clusters (High-High: high-value spatial clusters or Low-Low: low-value spatial clusters) and a low negative Z score represented the presence of spatial outliers (High-Low: high values surrounded with low values or Low-High: Low values surrounded with high values) [19,22]. Both Global Moran’s I and LISA were performed by ArcGIS software. Finally, Kulldorff’s space-time scan statistic (SaTScan software, version 9.5) was used to detect the location of possible high-risk space-time clusters for monthly scrub typhus cases from 2012 to 2020 at the county level. Clusters were scanned by the variable-sized moving window around the centroid of each county group[22,23].The null hypothesis assumed that the relative risk (RR) of the incidence was the same within the window as compared with outside. The difference of the incidence inside and outside the windows was evaluated by the Log Likelihood Ratio (LLR):

$$LLR=\log\{{\left(c/n\right)}^{c}{\left[\frac{C-c}{C-n}\right]}^{\left(C-c\right)}\}$$

Where C denotes the total number of cases; c is the number of observed cases inside the window; n is the number of expected cases inside the window. The window with largest LLR value is defined as the primary cluster and other windows with statistically significant LLR values are considered as secondary clusters [24]. Statistical significance (P-value<0.001) was evaluated through Monte Carlo simulation after 999 replications. In this study, according to the county-level scrub typhus cases, demographic data and geographical data, elliptic scan windows were selected to fit discrete Poisson models. The maximum spatial cluster size was set to 10% of the population at risk in the spatial window and a maximum temporal cluster size of 20% of the study period in the temporal window [10,18,25]. All the spatiotemporal clusters were mapped using ArcGIS version.

### Comparative Analysis of the demographic characteristics within and outside Clusters

To decide whether the differences in incidence are due to diverse demographic characteristics within and outside clusters, comparative analyses were conducted between high-risk counties (spatiotemporal clusters detected by space-time scan statistic) and low-risk counties (not included in the spatiotemporal clusters) using the χ^2^ test or the Z-test by SPSS software [17,22].

## Results

### Descriptive analysis

A total of 11,859 scrub typhus cases were reported from 87 counties from January 2012 to December 2020 in Fujian province (Table 1). Of these, 5,988 cases (50.49%) were males that were reported slightly more frequently than females with 5,871 cases (49.51%). There was significant sexual difference in scrub typhus incidence in 2014 with more female cases (χ^2^ = 6.250, P = 0.012) while 2018 is just the reverse in which the annual incidence rate was higher in males (4.87/100,000) compared to females (4.30/100,000) (χ^2^ = 6.843, P = 0.009). Our results also indicated that the greatest number of cases were observed in the age group of 50–59 years, which accounted for 28.33% of the total reported cases. Similarly, the high top 3 incidence rates occurred in the age groups of 50–59, 60–69, and 70–79 with the annual incidence of 7.26/100,000, 9.48/100,000, and 8.97/100,000, respectively.

**Table 1:** Annual number of scrub typhus cases and incidence rate by sex, age and occupation and the proportion of classified cases in Fujian Province, 2012–2020.

Characteristics		Number of cases (per 100 000 people)									Total	Mean annual incidence
		2012	2013	2014	2015	2016	2017	2018	2019	2020		
Sex	Male	421(2.18)	615(3.17)	600(3.10)	654(3.36)	761(3.86)	728(3.65)	981(4.87)	543(2.69)	685(3.19)	5988	3.34
	Female	445(2.44)	651(3.55)	667(3.57)	653(3.46)	750(3.94)	675(3.53)	828(4.30)	557(2.85)	645(3.21)	5871	3.43
Age	0–9	24(0.57)	29(0.68)	34(0.79)	27(0.61)	30(0.67)	35(0.77)	26(0.56)	12(0.25)	12(0.24)	229	0.57
	10–19	13(0.33)	21(0.56)	29(0.79)	30(0.84)	23(0.64)	19(0.53)	13(0.35)	14(0.37)	9(0.23)	171	0.51
	20–29	45(0.62)	62(0.87)	74(1.06)	67(0.98)	67(1.02)	60(0.97)	76(1.31)	38(0.72)	29(0.52)	518	0.9
	30–39	87(1.34)	116(1.81)	107(1.67)	104(1.62)	113(1.75)	97(1.46)	148(2.17)	86(1.21)	90(1.21)	948	1.58
	40–49	213(3.10)	292(4.23)	270(3.83)	282(3.97)	310(4.33)	263(3.62)	359(5.00)	205(2.89)	237(3.19)	2431	3.8
	50–59	226(5.44)	350(7.80)	354(7.60)	359(7.35)	469(9.12)	370(7.01)	509(9.11)	308(5.20)	415(6.70)	3360	7.26
	60–69	153(6.03)	254(9.47)	258(8.98)	298(9.67)	320(9.89)	367(10.57)	489(13.44)	300(8.11)	356(9.21)	2795	9.48
	70–79	90(6.13)	124(8.53)	115(7.93)	111(7.75)	149(10.37)	158(11.13)	158(11.07)	118(7.96)	153(9.87)	1176	8.97
	80+	15(2.49)	18(2.89)	26(4.14)	29(4.58)	30(4.64)	34(5.21)	31(4.77)	19(3.01)	29(4.39)	231	4.01
Occupation	Farmers	553	868	805	953	1099	1051	1377	794	1021	8521	
	Personnel living at home	77	133	171	117	179	146	186	120	140	1269	
	Workers	19	34	37	18	33	23	47	33	33	277	
	Pupils	14	26	30	20	20	14	14	16	8	162	
	others	160	99	120	98	117	120	119	80	85	998	
Case	Clinical	597	899	809	1028	1237	1144	1514	857	1104	9189	
classification	Confirmed	123	171	302	147	125	111	115	108	109	1311	
	Suspected	146	196	156	132	149	148	180	135	117	1359	

In regard to occupation, the largest proportion of scrub typhus cases is identified in farmers increasing from 67.19% in 2012 to 79.33% in 2020. Whereas, among the non-agricultural groups, most cases (11.30%) were predominantly attributed to the personnel living at home including homemaking service attendants, unemployed people and retirees, followed by workers (2.47%) and pupils (1.44%). Furthermore, 9,189 cases (77.49%) were reported as clinical cases, 1,311 (11.05%) of cases as confirmed cases, and 1,359 (11.46%) cases were recorded as suspected cases. The proportion of clinical cases towards the total cases over time from 68.94% in 2012 to 83.01% in 2020.

### Time-series analyses for scrub typhus

**Fig 1:**
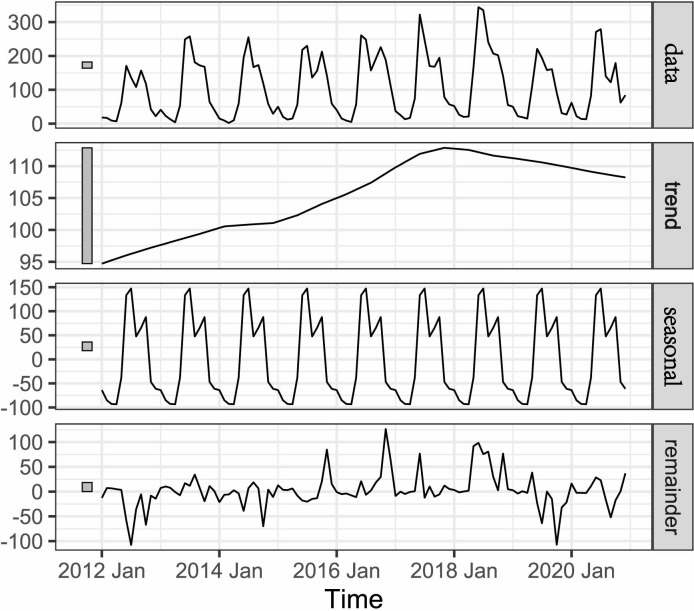
Temporal decomposition of scrub typhus cases of Fujian Province, January 2012–December 2020.

**Fig 2:**
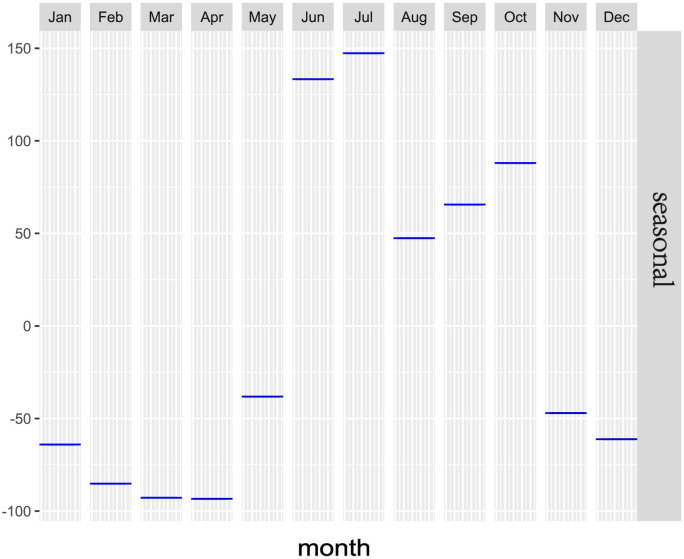
Seasonal subsequence from time-series analysis of reported monthly scrub typhus cases in Fujian Province, 2012–2020.

Based on the STL of temporal analysis, the monthly and annual variations of scrub typhus incidence are displayed to explore the whole trend and the seasonal dynamic during the study period (Fig 1). The maximum annual incidence was 4.59/100,000 in 2018 almost 2 times higher than the minimum reported 2.31/100,000 in 2012 (χ^2^ = 286.966, P<0.001), which indicated there was a rhythmic vibration in the raw incidence from 2012 to2020. Our analysis identified a non-linear trend for overall incidence in Fujian province with unimodal distribution which suggested an initial rapid increase from 2012 followed by a large peak in 2018 and a slight decline then onwards. The seasonal epidemic pattern that showed a bi-peak seasonality characteristic annually was presented (Fig 2) assuming that the seasonal effects each year are fixed when decomposing time series. The monthly incidence began to increase markedly in May with the primary peak happening in July and then diminished throughout August before reaching the second peak in October. At last, the disease returned to low and constant levels of transmission risk in the winter and spring months of November through April. In fact, scrub typhus cases occurred throughout the year but two seasonal peaks in June-July and September-October accounted for 37.38% and 25.45% of the total cases respectively. The annual increase range of the overall incidence was much smaller than seasonal variations which reflected in some large negative values in the seasonal component.

### Spatial distribution analyses for scrub typhus

**Fig 3:**
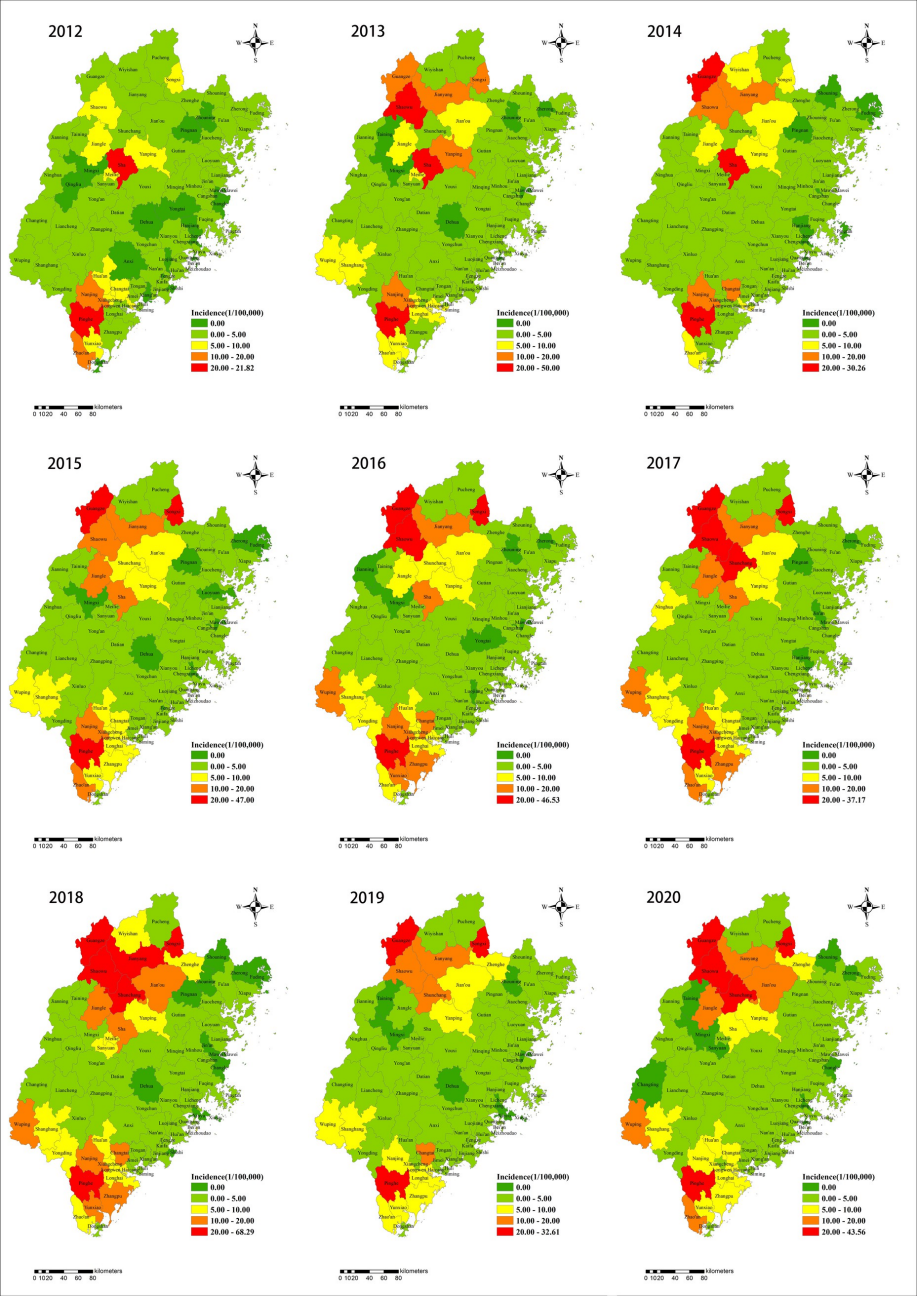
Spatial distributions in the annual incidence of scrub typhus in Fujian Province during 2012–2020. The base layer of the map was obtained from Resource and Environment Science and Data Center (https://www.resdc.cn/Datalist1.aspx?FieldTyepID=7,1).

The annualized incidence rate of scrub typhus in Fujian province was highly variable at the county level ranging from zero cases to 68.29 cases per 100000 residents. Our study demonstrates that the highest reported incidence rate was primarily observed in the two prevalent regions. One of the prevalent regions was concentratedly located in the north part of Fujian province including counties like Sha, Yanping, etc. And the other prevalent region was located in the southern counties containing Pinghe, Zhao’an, etc. (Fig 3) Annual incidence rates in northern region varies from 7.67/100,000 in 2012 to 26.21/100,000 in 2018 and in the south region the incidence ranges from 8.75/100,000 in 2014 to 17.54/100,000 in 2018. Among the 87 counties with reported cases of scrub typhus in the past 9 years, the accumulative case number occurred 3,172 cases in the north region and 4,049 cases in the south region, respectively.

### Spatial autocorrelation analyses for scrub typhus

The global spatial autocorrelation analysis for the incidence of scrub typhus at the county level in Fujian province during the study period revealed the significant positive results of spatial autocorrelation indicating that the geographical distribution of reported cases in Fujian province were clustered. The values of Global Moran’s I on the basis of statistical significance ranging from 0.258 to 0.471 demonstrated that the high or low scrub typhus incidence had strong spatial aggregation feature (Table 2).

**Table 2:** Yearly spatial autocorrelation analysis on scrub typhus incidence in Fujian Province, 2012–2020.

Year	Moran’s *I*	Z-Score	P-value
2012	0.423	5.424	<0.001
2013	0.371	5.233	<0.001
2014	0.369	4.788	<0.001
2015	0.258	3.487	<0.001
2016	0.43	5.631	<0.001
2017	0.437	5.506	<0.001
2018	0.471	6.069	<0.001
2019	0.383	5.017	<0.001
2020	0.44	5.56	<0.001

**Fig 4:**
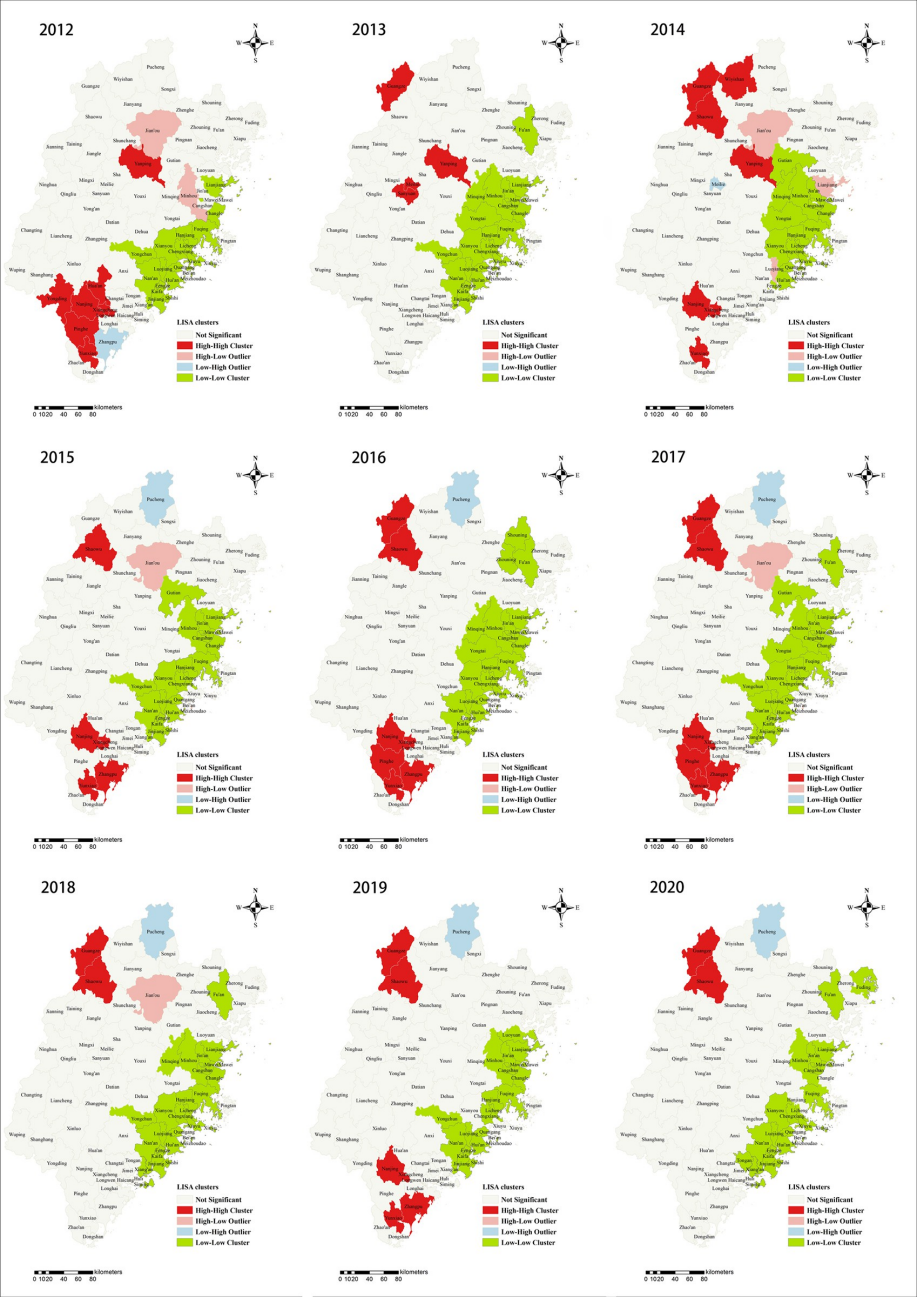
Yearly Local Indicators of Spatial Association (LISA) cluster maps for scrub typhus incidence in Fujian Province, 2012–2020. LISA spatial cluster map shows the center of the cluster in color. H-H indicates a statistically significant cluster of high scrub typhus incidence values; H-L represents high scrub typhus incidence values surrounded with low incidence values; L-H represents low scrub typhus incidence values surrounded with high incidence values. The base layer of the map was obtained from Resource and Environment Science and Data Center (https://www.resdc.cn/Datalist1.aspx?FieldTyepID=7,1).

LISA analysis was performed to identify significant hotspots (High-High), cold spots (Low-Low), and outliers (High-Low and Low-High) of scrub typhus transmission in Fujian province from 2012 to 2020 (Fig 4). High–High cluster areas were originally concentrated in the southern counties like Pinghe, Nanjing and Yunxiao and the central part of Fujian such as Yanping county. Although the hotspot in the south experienced a brief disappearance in 2013, it then gradually expanded to surrounding counties and developed into a large and contiguous geographic region of scrub typhus incidence. However, a shift of the hotspot’s location was observed and a relatively independent and stable High-High cluster occurred in northern Fujian including Guangze and Shaowu counties after 2013.

### Spatiotemporal clusters analysis

**Fig 5:**
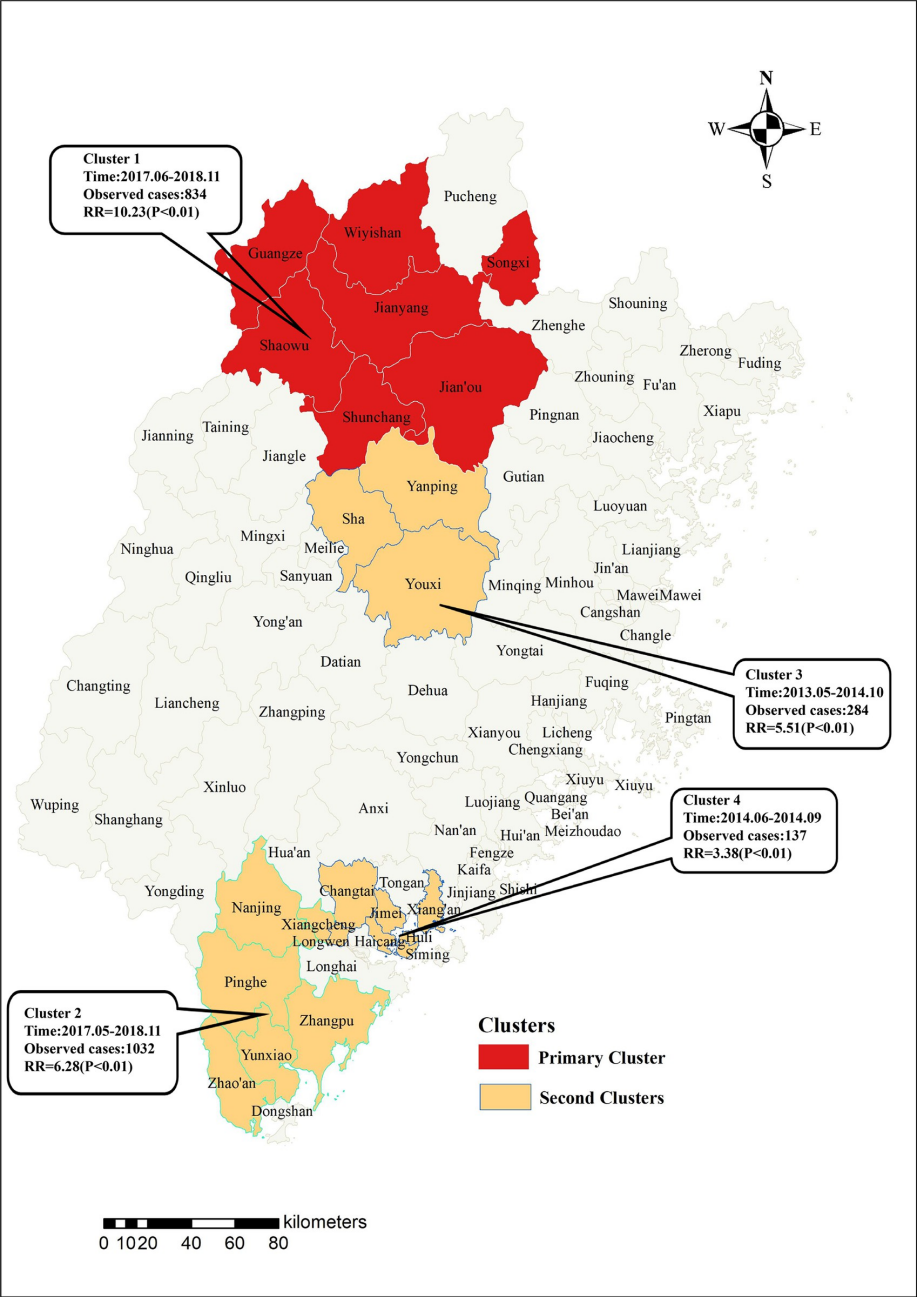
Spatiotemporal clusters of scrub typhus cases at the county level across the period of 2012–2020 in Fujian Province. The base layer of the map was obtained from Resource and Environment Science and Data Center (https://www.resdc.cn/Datalist1.aspx?FieldTyepID=7,1).

Using Kulldorff’s space-time scan statistic to detect spatiotemporal clusters across the study period, the primary cluster occurred in northern Fujian province including 7 counties in Nanping prefecture with a radius of 61.15 km (Fig 5). The time frame of the primary cluster was between June 2017 and November 2018 and the relative risk (RR) of scrub typhus infection for people inside the cluster was 10.23 (LLR = 1161.36, p<0.001) (Table 3). More importantly, the primary cluster, namely cluster 1 hereinafter mentioned only comprised 4.40% of the total population, whereas accounted for 27.88% of the total cases during the clustering time (Table 4). Besides, there were three significant secondary clusters (i.e., cluster 2, 3 and 4) identified which were principally distributed in 6 counties of Zhangzhou prefecture from May 2017 to November 2018, 3 counties in the middle part of Fujian province from May 2013 to October 2014 and 7 counties in the border region of Xiamen prefecture and Zhangzhou prefecture from June to September 2014, separately (Table 3).

**Table 3:** Spatiotemporal clusters of scrub typhus detected using Kulldorff’s space-time scan statistic in Fujian, 2012–2020*.

Clusters	E_Minor (Km)	E_Major (Km)	E_Anger (°C)	E_Shape	Time Frame	No.Counties	No.Obs	No.Exp	LLR	RR
@1	61	61	0	1	2017/6-2018/11	7	834	87	1161.36	10.23
#2	45	45	0	1	2017/5-2018/11	6	1032	177	994.79	6.28
3	29	59	-60	2	2013/5-2014/10	3	284	53	249.71	5.51
4	25	37	-45	1.5	2014/6-2014/9	7	137	41	69.84	3.38

*Significant clusters with P<0.01; @1: Primary cluster; #2–4: Secondary clusters; E_Minor: Semi-minor axis of ellipse; E_Major: Semimajor axis of ellipse; E_Angle: the angle between the horizontal line and the semimajor axis of the ellipse; E_Shape: E_Major/E_Minor; No.Counties: number of counties within clusters; No.Obs: number of observed cases; No.Exp: number of expected cases; LLR: log likelihood ratio; RR: relative risk for the cluster compared with the rest of the country.

**Table 4:** Scrub typhus incidence rate, proportion of population and cases in spatiotemporal clusters detected using Kulldorff’s space-time scan statistic in Fujian Province, 2012–2020*.

Clusters	Time Frame	Incidence (1/100,000)	Population (%)	Case (%)
※1	2017/6-2018/11	31.90	4.40	27.88
#2	2017/5-2018/11	19.40	8.42	33.67
3	2013/5-2014/10	18.00	2.71	12.97
4	2014/6-2014/9	11.20	9.95	17.34

*Significant clusters with P<0.01

※1: Primary cluster

#2–4: Secondary clusters; %: population or cases in cluster accounted for the total during the clustering time.

### Comparative demographic characteristics within and outside spatiotemporal clusters

Males accounted for the majority of cases in high-risk areas contrary to low-risk counties (Table 5). Compared with low-risk regions, the primary cluster, cluster 2 and cluster 3 had a higher proportion of males with a statistically significant difference (χ^2^ = 43.487, p < 0.001). With regard to the difference in the age distribution of scrub typhus cases, it was younger in cluster 2 and 4 than in the low-risk counties (Z = 6.255, p < 0.001), but it was older in clusters 3 (Z = -3.8, p < 0.001). The majority of reported scrub typhus cases occurred in the farmers and the concentration of high-risk areas was significantly higher (χ^2^>41.835, p < 0.001). Additionally, scrub typhus cases from the cluster 1 and cluster 3 had fewer days from illness onset to diagnosis with a median of nearly 4 days (Z = 4.493, p < 0.001) (Table 5).

**Table 5:** Comparison of characteristics of scrub typhus cases between high-risk counties and low-risk counties identified by Kulldorff’s spatiotemporal scan statistic in Fujian Province, 2012–2020.

Variables		High-risk counties				Low-risk counties
		Cluster 1	Cluster 2	Cluster 3	Cluster 4	
Sex	Male (%)	1356(58.02%) *	1897(50.17%) *	414(50.99%) *	525(49.39%)	1784(46.35%)
	Female (%)	981(41.98%)	1884(49.83%)	398(49.01%)	538(50.61%)	2065(53.65%)
Age	Median (Interquartile range)	55(46–64)	54(45–62) #	58(47–66) *	49(36–59) #	55(46–64)
Occupation	Farmers (%)	1837(78.61%) *	3223(85.24%) *	617(75.99%) *	365(34.34%) #	2470(64.17%)
	Not farmers (%)	591(21.39%)	616(14.76%)	195(24.01%)	698(65.66%)	1379(35.83%)
Days from illness onset to diagnosis	Median (Interquartile range)	5(2–10) #	5(2–10)	3(0–7) #	6(2–10)	6(2–11)

# Significant difference with lower value in high-risk counties compared with low-risk counties (χ2 test or Z test with p<0.05)

* Significant difference with higher value in high-risk counties compared with low-risk counties (χ2 test or Z test with p<0.05)

## Discussion

This study described the epidemiological characteristics and spatiotemporal patterns of scrub typhus in Fujian province using the completed and systematic dataset of monthly scrub typhus, which provided a good basis for understanding the epidemiological characteristics of the disease. Incidence data suggested that middle aged and elderly people were more prone to infection and two factors may contributed to this result. There is some evidence showing that the rapid change in the demographic structure happened in rural areas of Fujian province, where many young men work as laborers to the metropolitan areas for higher incomes, leaving their parents stay in the hometown [26–28]. The poor sanitary conditions and terrible living environment of rural areas are more susceptible to the dangers of disease probably because mice or other rodents carrying chigger vector are more common in such a place. Thus, chances are that rural left-behind people are more apt to come into contact with potential vector species outdoors and secondly these people often have lack of knowledge and practices on the prevention measures with low immunity and weak resistance resulting in the increased risk of infection [29,30].

In terms of occupations, farmers are also a high-risk group in Fujian province consistent with surveys conducted in neighboring provinces like the provinces of Guangdong, Zhejiang and Taiwan in China due to increased exposure opportunities to pathogen-carrying chigger mites and insufficient protection during cultivation, harvesting or other field agriculture activities [16,24,31,32]. More interestingly, we observed the growing proportion of homemaking service attendants, the unemployed and the retired in entire trend which could be associated with their more leisure time spared for outdoor recreational activities such as walking in parks. It has been reported in the literature that the eco-friendly trend of having more natural parkland and gardens within the city may create more suitable habitats for vectors and rodent hosts [33–35].

Another main outcome from this study is the successful qualitative investigation of the variation in reported incidence of scrub typhus on different time scales in Fujian province. Overall, the incidence rate of the disease showed an upward long-term trend between 2012 and 2020, and the highest annual incidence peaked in 2018. Such a steady increase can be explained in natural and socioeconomic perspectives. The current unprecedented global warming may play a specific role in the rising incidence of scrub typhus as the strong correlation between temperature and incidence was found in previous study from Guangzhou where the weather pattern is similar to Fujian [36]. In recent years, because the Fujian Provincial People’s Government actively respond to national policies and vigorously practice the construction of ecological civilization such as the recovery of forests and wetlands, the banning of the traditional burning of straw within rural areas and the reduction in industrial land use, a suitable setting for the growth of the rodent and chigger mite populations was produced increasing the possibility of human exposure and the formation of new natural foci for scrub typhus in previously unaffected areas[13]. For example, the construction of the first national ecological civilization pilot zone was approved in 2016 leading to the incidence rising until its highest point in 2018.

Seasonal decomposition analysis indicated that local acquired scrub typhus cases were highest in summer (June-July), followed by autumn (September-October). We believed that the seasonal pattern was influenced by the vector cycles and *Leptotrombidium deliense* is the major mite species and main vector is in the south of the Yangtze River in China [13,37,38]. The key determinants of scrub typhus transmission were temperature, relative humidity and precipitation and therefore, climatic conditions during June-October and abundant food in the harvest season might be most appropriate to the rodent host of *Leptotrombidium delicense* [38–41]. As a result, the peak incidence of scrub typhus coincides with the seasonal peak of *Leptotrombidium deliense*, a chigger that naturally harbors *O*. *tsutsugamushi* and can infest the rodents and humans [42].

There was the remarkable variation in the spatiotemporal distribution of scrub typhus cases in Fujian province, with most high-risk counties mainly concentrated in the Nanping and Zhangzhou prefecture and the geographical range of scrub typhus transmission increased over the study period, indicating that scrub typhus still remains a crucial public health problem. Both LISA and spatial distribution analysis detected similar changes and areas with high incidence appeared to shift from the south to the north mirroring the nationwide trends [10,23,43]. High-high spots which suggested that natural tsutsugamushi foci were forming here revealed the contribution of geography to the number of reported scrub typhus cases presumably in relation to elevated or mountainous region [44]. The clustering results demonstrated that the primary cluster was located in Nanping City from June 2017 to November 2018, which was consistent with the spatio-temporal combination of high incidence time and extended high-risk region identified above.

One of possible factors leading to the expansion of the epidemic zones and the shift of major foci is that secondary vegetation areas where chiggers and small mammal hosts are more abundant arose [13,45]. The mountainous area of Fujian province suffers from flood and other natural disasters and the ecological environment was gradually destroyed at first until Fujian provincial government has placed a more prominent strategic position in strengthening the capacity to mitigate the growing threat of natural disasters in the ecological circumstances [46]. Nevertheless, the generation of secondary vegetation, which helps provide moist terrain essential for chigger survival, was very beneficial to the formation of tsutsugamushi foci especially when the original vegetation of the mountainous foci recently recovered from natural hazards [45]. Additionally, due to the requirement of building a resource-conserving and environment-friendly society in accordance with the outline of the 12th Five-Year Plan for National Economic and Social Development of China, it is encouraged to promote the intensive and economical use of land and improve the use efficiency of cultivated land for agricultural modernization. Crop sown areas have decreased significantly and converting farmland to forest has become common in Fujian province [47,48]. Subsequently, partial abandonment of agricultural cultivation creates favorable habitats for trombiculid mites because crop remnants and natural vegetative regrowth provide the food and shelter necessary for proliferation of rodent hosts. This kind of conditions frequently occur in transitional land cover habitats, such as less populated forest edges or croplands left untended after agricultural practices [45]. In conclusion, habitat complexity and diversity of land cover are important drivers of the emergence of scrub typhus.

Despite our research brings important new knowledge on the epidemiological characteristics of scrub typhus in Fujian province, this study was subject to two specific limitations when interpreting our findings. Firstly, bias inevitably exists in this study due to the passive nature of the surveillance system [13]. Data quality may be influenced by the availability of diagnostic technology and underreporting. Only cases who sought medical care in hospital were reported to local CDC and used for analysis, therefore the under report of clinical cases or subclinical infection who recovered without healthcare were missed from the current analysis [37]. Meanwhile, diagnostic capabilities for scrub typhus at district hospitals are limited because of inability to perform confirmatory assays. As a result, the early and reliable diagnosis becomes inadequate and clinician’s awareness to expand their suspicion as differential diagnosis is restricted. Hence the number of reported cases was much smaller than the actual number of cases which are misdiagnosed, affecting the comparisons of incidence and the burden of scrub typhus estimated in this study is likely to be a gross underestimate of the true disease burden [49]. Secondly, the study did not address disease transmission models or cause and effect relationships. Crucial factors, including society, economy, geography and climate, all of which might be associated with the incidence heterogeneity, were not obtained for the current analysis for the moment.

In summary, this study analyzed the spatiotemporal dynamics of scrub typhus comprehensively and detailedly in Fujian province from 2012 to 2020. Our study revealed that a geographical and seasonal variation existed in Fujian province, and the high-risk areas mainly occurred in Fujian’s southern hilly region, expanding to the counties of Fujian’s northern mountainous areas and southeast of the Wuyi Mountains. In addition to contributing to the scientific advancement of scrub typhus epidemiology in Fujian province, the results from our study also provide pivotal evidence to health authorities, policy-makers and public health practitioners and other service providers to devise feasible strategies and issue targeted guidelines. For example, future land use policy decision-making should ensure long-standing potential public health outcomes [45]. Knowledge of the geographical distribution and burden of scrub typhus is vital to determine the optimal allocation of limited resources necessary for disease prevention and control. At the same time, physicians should raise awareness of nonspecific fever symptoms among the residents in high-risk areas so as to diagnose scrub typhus patients earlier and more precisely [43]. The study is beneficial for awareness about the neglected but serious public health issue. Health promotion strategies, such as educating personal protection methods and improving personal hygiene and environmental sanitation in collaboration with relevant government departments, are highly recommended [50]. The relationships between the potential influential factors in geographical, climatic and environmental aspects and scrub typhus incidence in Fujian province would be further explored in the future.
